# Successful pregnancy in pulmonary arterial hypertension associated with systemic lupus erythematosus: a case report

**DOI:** 10.4076/1752-1947-3-7255

**Published:** 2009-06-09

**Authors:** Michael Streit, Rudolf Speich, Manuel Fischler, Silvia Ulrich

**Affiliations:** 1 Department of Internal Medicine, University Hospital of Zurich, Raemistrasse 100, 8091 Zurich, Switzerland

## Abstract

**Introduction:**

Pulmonary arterial hypertension is a complication of systemic lupus erythematosus. Mortality in pregnant patients with pulmonary arterial hypertension related to connective tissue disease is as high as 56%. The authors report the first case of a successful maternal-fetal outcome in a pregnant patient with systemic lupus erythematosus-associated pulmonary arterial hypertension treated with sildenafil and inhaled iloprost during pregnancy and until several weeks after caesarean section.

**Case presentation:**

The case presented is of a 29-year-old woman with systemic lupus erythematosus and associated severe pulmonary arterial hypertension. Vasodilator therapy with bosentan and sildenafil, immunosuppressive therapy with prednisone, hydroxychloroquine and azathioprine and oral anticoagulation (phenprocoumon) had normalized her right ventricular over right atrial pressure when she was diagnosed in her 5th week of pregnancy. The teratogenic drugs bosentan and phenprocoumon were stopped, the latter replaced by low molecular weight heparin. During the 35th week, a slight increase in pulmonary pressure was found. Therapy with inhaled iloprost was established. A caesarean section was performed in the 37th week and a healthy baby was delivered. The patient remained stable until 11 weeks after delivery, when an increase in right ventricular over right atrial pressure was noted. Bosentan was reintroduced and prednisone and azathioprine doses were increased. The patient has remained stable until the present time.

**Conclusion:**

Pulmonary arterial hypertension has been considered a contraindication for pregnancy. Novel vasodilator therapy, combined with immunosuppressants in this patient with systemic lupus erythematosus, may "cure" pulmonary arterial hypertension and permit pregnancy with successful outcome. However, postpartum exacerbation of systemic lupus erythematosus and pulmonary arterial hypertension have to be considered.

## Introduction

Systemic lupus erythematosus (SLE) occurs most often in the reproductive years of female patients [1]. The percentage of pulmonary arterial hypertension (PAH) in SLE has been reported to range from 0.5% to 14% [2]. PAH is aggravated by physiological changes associated with pregnancy [3]. Mortality in pregnant patients with PAH related to connective tissue disease is as high as 56% [4]. Typically, patients die after delivery due to acute right ventricular failure [4]. Herein we report the first case of a successful maternal-fetal outcome in a pregnant patient with SLE-associated PAH treated with sildenafil and inhaled iloprost during pregnancy and until several weeks after caesarean section.

## Case presentation

A 29-year-old woman had been diagnosed with SLE according to the American College of Rheumatology criteria 6 years earlier due to malar rash, photosensitivity, arthritis of the finger joints, wrists and knees, pleural effusion and abnormal titers of the antinuclear and antinative DNA antibodies. Treatment consisted of prednisone as needed and hydroxychloroquine (200 mg/day). Five years after the initial diagnosis, the patient developed exertional dyspnea. Severe PAH was diagnosed by echocardiography and confirmed by pulmonary arterial catheterization (PAC) (Table 1).

**Table 1:** Summary of recorded physiology

			Pregnancy				
	Diagnosis PAH	3 months later	5^th^ week	20^th^ week	35^th^ week	Delivery	3 months postpartum
NYHA	IV	III	III	III	III	III	IV
6-MWT m	229	423	454	396	276		392
RVSP mmHg (Echo)	69		normal	normal	40		57
SPAP mmHg	72	57				45	
MPAP mmHg	52	35				21	
PAOP mmHg	10	8				7	
CI L/min/m^2^	1.3	3.4				4.3	
PVR dyn.sec.cm^−5^	1580	410				250	
mVO_2_%	35	67				69	
SLEDAI [14]	17	2	2			14	18
Phenprocoumon (INR)		2.1	2.2				2.1
LMWH (Dalteparin E/day)				10,000	10,000		
Prednisone (mg/day)	50	30	10	25	25	50	50
Hydroxychloroquine (mg/day)	200	200	200	200	200	200	200
Cyclophosphamide (mg/day)	100	100					
Azathioprine (mg/day)			100	100	100	100	150
Bosentan (mg/day)	250	250					250
Sildenafil (mg/day)	150	150	150	150	150	150	150
Inhaled iloprost (μg/day)					50	100	

NYHA, New York Heart Association; 6-MWT, 6 minute walking test distance; RVSP, echocardiographic systolic right ventricular pressure over right atrial pressure; hemodynamic assessments by right heart catheterization: SPAP, systolic pulmonary arterial pressure; MPAP, mean pulmonary arterial pressure; PAOP, pulmonary arterial occlusive pressure; CI, cardiac index; PVR, pulmonary vascular resistance; mVO_2_, mixed venous oxygen saturation; SLEDAI, systemic lupus erythematosus disease activity index; LMWH, low molecular weight heparin.

Oral anticoagulation with phenprocoumon and treatment with bosentan (125 mg bid) and sildenafil (50 mg tid) was initiated. Cyclophosphamide (100 mg qd) was added to the SLE therapy for 4 months and thereafter replaced by azathioprine (100 mg qd). The patient's clinical status improved significantly and repeated PAC 3 months later showed a significant decrease in mean pulmonary arterial pressure (MPAP) with a concomitant increase in cardiac index (CI) resulting in a decreased pulmonary vascular resistance (PVR) (Table 1). Echocardiography performed after a further 8 months under a stable clinical condition showed completely normalized right heart dimensions and pulmonary arterial pressure. One week later, pregnancy at the 5th week of gestation was diagnosed, despite repeated advice to practice birth control and insistent information about the maternal and fetal risks of pregnancy in SLE-associated PAH as well as the teratogenic potential of bosentan and phenprocoumon. Since a termination of the pregnancy was out of the question for the patient, bosentan and phenprocoumon were immediately stopped and low molecular weight heparin (LMWH) was started. Sildenafil, azathioprine, and hydroxychloroquine were left unchanged whereas prednisone was augmented to 25 mg daily to prevent SLE relapse. The patient was followed fortnightly by an interdisciplinary team and repeated clinical and echocardiographic examinations remained stable until the 35th week of gestation. Intrauterine fetal growth assessed sonographically was normal at all times.

At 35 weeks however, echocardiography showed an elevated right ventricular systolic pressure (RVSP) of 40 mmHg above the right atrial pressure. The patient was hospitalized and treatment with inhaled iloprost was established 5 to 6 times daily at an initial daily dose of 50 μg, increasing to 100 μg within 1 week. A caesarean section was scheduled for week 37. Hemodynamic assessment with PAC before the operation showed a MPAP between 20 and 26 mmHg with a CI around 4-5 L/min/m^2^. A healthy female infant weighing 2760 g with Apgar scores of 8, 9 and 10 at 1, 5 and 10 minutes, respectively, was delivered via caesarean section under epidural anesthesia. The patient remained stable throughout the operation. The PAC was continued during 24 hours postoperatively showing stable pulmonary hemodynamics. Prednisone was augmented to 50 mg qd on the day of delivery and then continued at a dose of 30 mg qd for the next 2 months to prevent postpartal relapse of SLE. Mother and child were discharged 10 days later under stable conditions. Breast-feeding was declined. Sildenafil, inhaled iloprost, azathioprine and hydroxychloroquine were continued, LMWH was replaced by phenprocoumon. Eleven weeks later, progressive dyspnea and fatigue developed and a relapse of SLE was diagnosed with reappearance of tender swollen joints in the fingers, wrists and knees, malar rash and pleuropericardial effusion. Echocardiography showed a rise in RVSP to 57 mmHg. Prednisone and azathioprine were augmented to 50 mg qd and 150 mg qd, respectively, and bosentan 125 mg bid was added to the established therapy. The patient remains in a stable condition and her daughter is thriving well.

## Discussion

This case demonstrates for the first time cure of SLE-associated PAH and successful pregnancy with combination immunosuppressive and vasodilator therapy.

In healthy women, physiological changes associated with pregnancy and puerperium pose a great challenge on the cardiovascular system. During this period, the CI increases by 30% to 50%, the blood volume by 50%, and oxygen consumption by 20% [4]. This significant increase in pulmonary blood flow is maintained at an unchanged MPAP resulting in a decreased PVR [5]. Immediately after delivery an autotransfusion of approximately 500 ml of blood from the involuting uterus occurs, leading to a further increase in maternal blood volume [3]. Patients with PAH have a limited ability to compensate for this increase in CI and blood volume due to their fixed resistance of the pulmonary vasculature. This may lead to acute or chronic right heart failure with the highest incidence of maternal mortality occurring during the first days after delivery [4]. No agreement exists concerning the influence of pregnancy on the course of SLE. The reported incidence of SLE flares during pregnancy ranges from 13% to 60%, leading some investigators to believe the flare rate is unchanged while others consider pregnancy as a time of vulnerability to increased disease activity [6,7]. Patients with inactive SLE at conception are less likely to experience flares during pregnancy [6]. Our patient had normalized her pulmonary artery pressure assessed by echocardiography just before pregnancy under intensive therapy. To what extent this was a prerequisite for the uneventful pregnancy remains unknown. From pathophysiological and clinical experience, it must be assumed that the pregnancy-related mortality will increase with the degree of PAH.

Patients with SLE-related antiphospholipid syndrome are at increased risk for spontaneous miscarriage. Antiphospholipid antibodies were not found in our patient. Another threat to fetal outcome in SLE patients is neonatal lupus, a transferred autoimmune syndrome occurring in babies born to mothers with anti-Ro antibodies, which may lead to complete heart block and isolated skin rash. Our patient did have anti-Ro antibodies, fortunately however, no signs of intrauterine or postpartal heart block were detected by repeated pulsed duplex echocardiography and no skin manifestations were present after delivery.

Medical therapy of patients with PAH has improved considerably in recent years. Available drugs focus on the main dysfunctional pathways known to date and include anticoagulation therapy, prostaglandin analogues for example, epoprostenol, iloprost, treprostinil; endothelin-1 receptor antagonists for example, bosentan; and phosphodiesterase 5 inhibitors for example, sildenafil. With respect to pregnancy, bosentan is contraindicated due to its teratogenic potential [8]. Anticoagulation therapy is generally recommended in PAH. As our patient improved considerably on anticoagulation, immunosuppressive therapy and newer vasodilator and antiproliferative agents and we did not know to what extent which of the measures was responsible for the amelioration of the patient, we decided to maintain the therapy as far as possible. We replaced the potentially teratogenic phenprocoumon with low molecular weight heparin but stopped bosentan. Recently, several reports of successful maternal and fetal outcome with intravenous and inhaled iloprost treatment at different stages of pregnancy and postpartum period have been published [9,10]. Nonetheless, cases of maternal deaths are still not unusual [9].

To the best of our knowledge, the use of sildenafil during pregnancy in patients with PAH has only been described in two patients with Eisenmenger's syndrome. One of them was a 22-year-old patient treated with sildenafil 150 mg/day during gestational weeks seven and eight. Sildenafil was discontinued thereafter for financial reasons but restarted at gestational week 31 after an acute exacerbation of PAH with a good outcome of mother and infant [11]. The second case reports a 23-year-old patient treated with bosentan in whom pregnancy was not diagnosed before 28 weeks of gestation. Sildenafil was added, and both drugs were continued until the premature termination of pregnancy via a caesarean section at week 30 because of maternal cardiopulmonary deterioration and decreased fetal movements. The delivered baby girl was healthy and without signs of bosentan teratogenicity [12].

Our patient with PAH not related to Eisenmenger's syndrome is the first treated with sildenafil during the entire pregnancy. Until today, nothing was known about the possible teratogenic effects of sildenafil. It inhibits phosphodiesterase type 5, an enzyme that metabolizes cyclic guanosine monophosphate (cGMP) thereby enhancing the cGMP mediated relaxation and growth inhibition of smooth muscle in the lung vasculature [13]. Vasodilative therapy with oral sildenafil controlled our patient's PAH well until gestational week 35. The relatively mild deterioration thereafter was successfully treated by adding inhaled iloprost. Sildenafil might have additional beneficial effects with respect to fetal growth retardation (FGR) in the context of pregnancies at risk, as this is well known in patients with PAH and SLE [14]. FGR is typically related to placental insufficiency due to reduced blood flow and increased resistance in the uterine arteries. *In vitro*, the incubation of sildenafil with small myometrial arteries from women with a history of FGR showed a decreased response to vasoconstrictors. This might suggest a potential beneficial effect of sildenafil on uteroplacental blood flow *in vivo* as well [15].

## Conclusion

This case strengthens our hypothesis and might place sildenafil as first line therapy for PAH in pregnancy. The combination of sildenafil with prostaglandin analogues for the management of disease exacerbations in SLE broadens the therapeutic armamentarium with potentially less side effects than the prolonged single use of prostaglandin analogues. However, sildenafil's true therapeutic potential during pregnancy awaits further clinical evaluation, and it has to be stressed that, despite most modern treatment options, the mortality rate of pregnancy in PAH remains high.

## Abbreviations

cGMP: cyclic guanosine monophosphate; CI: cardiac index; FGR: fetal growth retardation; LMWH: low molecular weight heparin; MPAP: mean pulmonary arterial pressure; PAC: pulmonary arterial catheterization; PAH: pulmonary arterial hypertension; PVR: pulmonary vascular resistance; RVSP: right ventricular systolic pressure over right atrial pressure; SLE: systemic lupus erythematosus.

## Consent

Written informed consent was obtained from the patient for publication of this case report. A copy of the written consent is available for review by the Editor-in-Chief of this journal.

## Competing interests

The authors declare that they have no competing interests.

## Authors' contributions

MS searched the literature and drafted the manuscript. MS, RS, MF and SU managed the clinical case of the patient. RS, MF and SU edited the manuscript. The final manuscript has been seen and approved by all the authors.
